# The impact of the Ebola virus disease (EVD) epidemic on agricultural production and livelihoods in Liberia

**DOI:** 10.1371/journal.pntd.0006580

**Published:** 2018-08-02

**Authors:** Tsegaye T. Gatiso, Isabel Ordaz-Németh, Trokon Grimes, Menladi Lormie, Clement Tweh, Hjalmar S. Kühl, Jessica Junker

**Affiliations:** 1 German Centre for Integrative Biodiversity Research (iDiv), Halle-Jena-Leipzig, Deutscher Platz 5E, Leipzig, Germany; 2 Max Planck Institute for Evolutionary Anthropology, Department of Primatology, Deutscher Platz 6, Leipzig, Germany; 3 Forestry Development Authority, Wheintown, Mount Barclay, Liberia; Institute for Disease Modeling, UNITED STATES

## Abstract

There is unequivocal evidence in the literature that epidemics adversely affect the livelihoods of individuals, households and communities. However, evidence in the literature is dominated by the socioeconomic impacts of HIV/AIDS and malaria, while evidence on the impact of the Ebola virus disease (EVD) on households’ livelihoods remains fragmented and scant. Our study investigates the effect of the EVD epidemic on the livelihoods of Liberian households using the Sustainable Livelihood Framework (SLF). The study also explores the effect of the EVD epidemic on agricultural production and productive efficiency of farm households using Spatial Stochastic Frontier Analysis (SSFA). We collected data from 623 households across Liberia in 2015, using a systematic random sampling design. Our results indicated that the annual income of sample households from communities where EVD occurred did not differ from the annual income of households from communities where EVD did not occur. Nonetheless, the majority of sample households reported a decrease in their income, compared to their income in the year before the survey. This suggests that the impact of the EVD epidemic might not only have been limited to communities directly affected by the epidemic, but also it may have indirectly affected communities in areas where EVD was not reported. We also found that the community-level incidence of EVD negatively affected crop production of farm households, which may have exacerbated the problem of food insecurity throughout the country. Moreover, we found that the EVD epidemic weakened the society’s trust in Liberian institutions. In a nutshell, our results highlight that epidemics, such as the recent EVD outbreak, may have long-lasting negative effects on the livelihoods of a society and their effect may extend beyond the communities directly affected by the epidemics. This means that the nation’s recovery from the impact of the epidemic would be more challenging, and the social and economic impacts of the epidemic may extend well beyond the end of the health crisis.

## Author summary

Epidemics such as HIV/AIDS, malaria and Ebola virus disease (EVD) may adversely impact the livelihoods of the society affected by the epidemics. Nonetheless, the mechanism behind the effects of the epidemics may differ depending on different factors, such as the transmission mechanisms, latency, and mortality rates associated with the diseases, which requires specific research to investigate the effect of each epidemic. In light of this, we analyzed the impact of the recent EVD epidemic on the agricultural production of farm households and its impact on the livelihoods of Liberian society. We collected data from 623 households throughout Liberia during the EVD crisis in 2014–2016, and found that there was no significant difference in the annual income of sample households from communities where EVD occurred and did not occur. Nonetheless, the majority of the sample households reported a decrease in their income compared to the year before our survey. We also found that the community level incidence of EVD had a significant negative effect on crop production of farm households, which might have exacerbated food insecurity in the country. Moreover, the EVD epidemic negatively affected the Liberian society’s trust in Liberian institutions. Our results underline that epidemics, like EVD, might have long-lasting negative effects on the livelihoods of a society, and they may have adverse effect beyond the communities directly affected by the epidemics.

## Introduction

There is a plethora of evidence in the literature that epidemics such as HIV/AIDS and malaria have profound implications for the livelihoods of the affected society. The impact of HIV/AIDS on livelihoods has been intensively investigated and there is universal consensus that HIV/AIDS adversely affects the livelihoods of individuals, households and communities [1–2], and has macro-level implications for poverty, economic growth, unemployment and political stability [3–7]. Similarly, malaria has been found to have a strong negative effect on the socioeconomic status of households [8–11]. In contrast, the socioeconomic impacts of the Ebola virus disease (EVD) epidemic, specifically the most recent and largest ever EVD epidemic recorded in West Africa from 2014–2016, have not been systematically analyzed. Due to the differences in transmission mechanisms, latency, and mortality rate between EVD and other infectious tropical diseases, such as HIV/AIDS and malaria [12–14], EVD outbreaks likely impact the livelihoods of a society differently. For example, EVD can wipe out an entire family or village within a relatively short period of time. In areas affected by EVD, economic activities may cease completely, as people no longer work on their fields, nor trade or even travel (because of check points and travel restrictions) [15]. HIV/AIDS infections and resultant mortalities, on the other hand, occur over a longer time period; and as such, their effects on livelihoods and the economy are more subtle at first. HIV/AIDS and malaria result in higher costs in terms of the opportunity cost of the time spent caring for the sick household member [16–18], medical expenses and, for the unlucky ones, funeral expenses. In contrast, EVD lowers livelihood outcomes by weakening the ability of the households to earn their living rather than by increasing the expenditure on the sick person and funeral ceremonies. For example, the medical costs of the EVD epidemic in Liberia were mostly covered by the government and the international community, as the epidemic presented a global health emergency. These and other disease-specific characteristics necessitate specific research to investigate the effect of different infectious diseases on the livelihood of the affected societies. In light of this, we analyze the impact of the recent EVD epidemic on the livelihoods of the Liberian society.

There are few studies that explored the impact of the EVD epidemic on the agricultural sector in Liberia [19–20]. These studies reported that the EVD epidemic negatively affected employment in the agricultural sector. At the peak of the epidemic, almost half of the country’s labor force was out of work [19–20]. Farmers were less likely to work on their farms during the EVD epidemic [19]. These studies found that most of the households returned to their farms during the survey, which was conducted from October, 21 to November, 7, 2014, and concluded that the impact of the EVD crisis on the agricultural sector may not have been as severe as predicted [19–20]. Nonetheless, these studies used phone surveys to collect data, which could have resulted in selection bias, as households without mobile phones were systematically excluded from the survey. In addition, the emphasis of the studies was focused on the impacts of the EVD epidemic on the employment in the agricultural sector. However, employment and whether or not households were working on their farm tell only part of the story. Even if households were working on their farms during the EVD epidemic, the productivity of their agricultural inputs, and hence their efficiency may have been compromised by the epidemic. Therefore, our study provides evidence on how the Ebola crisis affected the efficiency of farm households, and the concomitant effects on agricultural production in Liberia. Moreover, our study explores the impact of the EVD epidemic on livelihoods of the Liberian society using the sustainable livelihood framework (SLF) [21–22].

The EVD epidemic affects the livelihoods of individuals, households and communities by weakening the household assets upon which the households’ ability to enhance their livelihood, depends [21]. These assets can broadly be categorized into five categories: physical capital (e.g., infrastructure, tools, equipment), human capital (e.g., knowledge and ability to work), financial capital (e.g., available stocks, access to financial services, regular inflows of money), social capital (e.g., networks for cooperation, trust, support) and natural capital (e.g., land, forests, water) [21–24]. Shocks, such as epidemics, that weaken some or all of these household assets, negatively affect livelihood [2, 25]. Therefore, a complete understanding of the effects of epidemics, such as EVD, on the livelihood of households requires the investigation of their influence on the assets owned by the households.

The EVD epidemic may have weakened the resource base of the Liberian society for various reasons. First, the incidence of the epidemic in the households and/or their communities may have weakened the different categories of assets owned by households directly [2, 24]. Second, measures taken by the Liberian government to contain the spread of the disease may have further dampened the households’ assets and affected their welfare [15, 26, 27]. For example, the government declared a state of emergency and established quarantine zones in most of the affected communities. Schools and markets were closed in several districts and communities. Restrictions on domestic and international travels were imposed [15, 19, 26, 27]. Thus, the mobility restrictions and complete closure of markets might have considerably hampered the livelihoods of individuals, households and communities by reducing their access to different livelihood assets [15, 26]. Third, fear of contracting the disease may have coerced people into avoiding social gatherings and participation in different activities and organizations, thereby weakening the social capital that the Liberian society possesses [27]. Furthermore, during crises, people may increasingly seek support from different social networks such as friends, families, the community, and the government, but the prospect of receiving the needed help may have been significantly hampered by an epidemic, which strains the social cohesion. Stigmatization of survivors of the disease may also contribute to the degradation of the social capital.

Our study uses a systematic nationwide random sampling design to explore the effect of the EVD epidemic on the livelihood assets possessed by Liberian households, and the livelihood outcomes they achieved during the EVD epidemic. In addition, we emphasize the effect of the epidemic on the agricultural sector. We are interested in the effect of the EVD epidemic on agricultural production for two main reasons. First, the agricultural sector plays a crucial role in Liberia and contributes more than 35% of the country’s GDP [28]. Moreover, the majority of Liberians live in rural areas (50%), and are primarily engaged in the agricultural sector to earn a living [20]. Almost 80% of rural households and 18% of urban households are agricultural households in Liberia [29]. Second, we are not aware of any study that examined the effect of the recent EVD epidemic on agricultural production in Liberia or elsewhere accounting for the productive efficiency of farm households.

## Materials and methods

### Study sites, sample selection and sources of data

**Fig 1:**
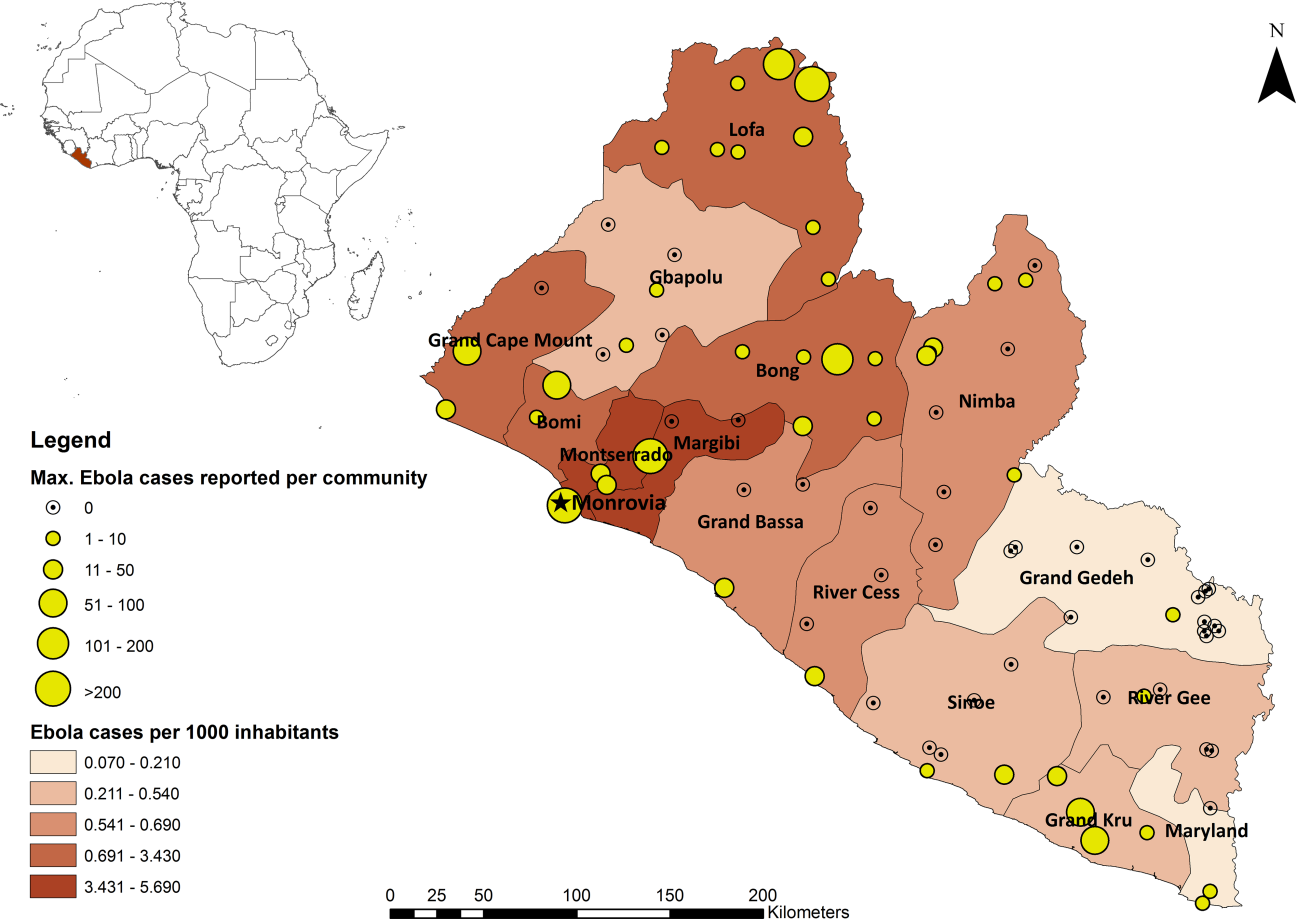
Map of Liberia showing county-level [33] and community-level EVD cases.

Liberia is one of the poorest countries in Africa with per capita GDP of $457.9 as of 2014 [30]. Nevertheless, after the end of the civil war in 2003, the country’s economy has been steadily growing. For instance, the economy grew by an annual growth rate of 8% in 2006 and 8.7% in 2013 [30]. Although most of the population lives in poverty (68.6% of the population lives on less than $1.9 a day), Liberia had one of the fastest growing economies in Africa over the past 10 years [31]. This gain of momentum in terms of macro-economic performance was disrupted in 2014 by the epidemic of EVD [32], which affected the country from 2014 to 2016 and resulted in the tragic loss of 4,809 lives (45% of reported cases) [33]. All 15 counties reported incidences of the disease, but the severity of the epidemic varied from place to place [33]. For example, based on the number of EVD cases per 1,000 inhabitants, counties such as Margibi, Montserrado, Grand Cape Mount, and Bomi were more severely affected by the epidemic than Grand Gedeh, River Gee, Sineo and Maryland [33] (Fig 1).

We conducted a systematic nationwide household survey from February to June, 2015. The survey was administered in person by trained Liberian enumerators. Sample households were randomly selected following a “random walk” procedure [e.g., 34, 35]. Starting from the center of a village/town/city (hereafter ‘interview location’), the enumerators walked in different directions and randomly selected households to be interviewed. We aimed at interviewing 5–10 households per location, depending on their size (i.e., more interviews in larger locations). The random walk technique was used to reduce non-response rates, as the enumerators would walk until they found enough households that were willing to participate in the interview. This method is particularly useful in sensitive times like the EVD crisis, when people may have been more reluctant to interact with strangers out of fear of contracting the EVD. Nonetheless, we acknowledge that in larger locations, the random walk method may have resulted in some biases, as households closer to the center of locations were more likely to be sampled than those living farther away from the centers.

Whenever possible, household heads were selected and interviewed. A total of 623 sample households were interviewed. We collected data through face-to-face interviews with sample households across Liberia using a questionnaire (S6 Appendix). We were granted permission to conduct the survey by Liberian authorities after careful evaluation of staff safety, data collection procedures and agreements on data sharing (see S7 Appendix). Additional data on EVD deaths and cases were obtained from the World Health Organization (WHO) and the Liberia Institute of Statistics and Geo-information Services (LISGIS).

### Conceptual framework: Sustainable Livelihoods Framework

To explore the impact of EVD on the livelihoods of Liberian society, we applied the Sustainable Livelihoods Framework (SLF) used by the Department for International Development-United Kingdom (DFID-UK) [21–22]. We used the SLF as it enables us to understand not only the effects of the EVD epidemic on livelihood outcomes, but also the mechanisms driving the effects of the epidemic [21–22]. Thus, our study provides important insight into policies aiming to avert or reduce the impact of future epidemics on livelihoods by addressing the important factors that drive these effects. Although there is a scarcity of studies that applied this framework to investigate the impact of EVD, several studies employed the SLF to explore the livelihood effects of other epidemics, mainly HIV/AIDS [1, 2].

Traditionally, the SLF has been applied to understand differential capabilities of rural families to cope with stresses or shocks [36] and their ability to achieve sustainable livelihoods. A livelihood is defined as a means of living, and the assets required to achieving it [21–22]. The different types of assets that a household needs to achieve better livelihood outcomes are broadly categorized as human, physical, financial, social and natural capitals [21, 22, 24]. Hence, the likelihood of a household to achieve improved livelihood outcomes (such as income, food security and others) depends on its access to different livelihood assets. The livelihood of a household is deemed sustainable when it copes with, and recovers from stresses without compromising the abilities of future generations [21]. Factors that weaken some or all of the livelihood assets of households, adversely affect their livelihood [2, 25]. Therefore, a complete understanding of the effect of shocks, such as epidemics, on the livelihood of households necessitates the investigation of their influence on the assets owned by the households. Employing the SLF, we investigate the impact of the EVD epidemic on different categories of assets possessed by Liberian households, and the livelihood outcomes they achieved during the EVD epidemic (i.e., in the 12 months preceding our survey). Here, we focused on the effect of the EVD epidemic on three categories of household capitals (natural, financial, and social capitals), and their total income and agricultural production. See Table 1 for the definition of the household assets included in our study.

**Table 1:** Categories of household assets included in the study and their definition.

Categories of household assets	Definition
Natural capital	A binary variable for uncultivated land owned by households
Financial Capital	1. Cash income from different sources 2. Livestock ownership in TLU
Social capital	1. A composite index for trust in one’s community 2. A composite index for trust in institutions

#### Computation of the livelihood outcome variables

We used total household income as an indicator of livelihood outcome. To measure this, we estimated the income of households from different sources such as farming, wage employment, remittance, aid, gifts in terms of gross margins, which is the difference between gross income and variable costs. For example, crop income for household *h* in terms of gross margins (*GM*_*c*,*h*_) is the total sum of the differences between the revenue obtained from each crop (*p*_*j*_*y*_*j*_) and variable costs incurred by the household to produce it (*c*_*j*_), and can be given as:

$${GM}_{c,h}=\sum_{j=1}^{m}{(p}_{j}y_{j}-c_{j})$$ (1)

where *p*_*j*_ is the price of the crop j; *y*_*j*_ is the total production of crop j, and *m* is the number of crops produced by household *h*.

The total annual income from other sources was computed in a similar manner by modifying Eq 1 and calculating the difference between total revenue/income from the specific source and variable costs associated with it (i.e., obtaining source specific GMs). Finally, we computed the total annual income of a household by aggregating the source specific *GM*s as given below:

$$Total\;household\;income=\sum_{k=1}^{K}{GM}_{k}$$ (1A)

Where *k* represents the number of sources of the household income

In our questionnaire, we explicitly asked respondents to report the amount of income they obtained from different sources and the associated costs in the 12 months prior to the survey (see S6 Appendix).

For farm households, we examined the effect of the EVD epidemic on agricultural production in addition to its effect on total household income. To investigate the effect of the EVD epidemic on crop production, we used the stochastic production frontier framework (SPFF) [37]. According to the SPFF, the agricultural output produced by farm households is influenced by deterministic factors (such as the quantity and quality of inputs used), and stochastic factors. The basic characteristic of this framework is that it decomposes the stochastic component of the production function into (1) a non-systematic stochastic component, which accounts for factors outside of the control of the farm household, and (2) a systematic stochastic term, which accounts for technical inefficiency of the farm household [37]. Technical efficiency is defined as the ability of a production unit (e.g., a firm, an individual, or a household) to produce the maximum output for a given level of inputs and technology [38].

Under the SPFF, the agricultural production function for a farm household *h* can be given as:

$$Y_{h}=f(X_{ih},\beta )exp(v_{h}-u_{h})$$ (2)

Where *Y*_*h*_ is the value of farm output of household *h* per hectare; *X*_*i*,*h*_ is a matrix of farm inputs used by a household *h* to produce *Y*_*h*_, and *β* is a vector of parameters to be estimated from the model; *v*_*h*_ is the stochastic term, which is independent and identically distributed with a mean of 0 and a constant variance $\sigma_{v}^{2}$ (i.e., $v_{h}∼N(0,\sigma_{v}^{2})$); and *u*_*h*_ represents a stochastic term which accounts for the technical inefficiency of a farm household *h*. *u*_*h*_ is independent and normally distributed with a mean *μ*_*h*_ and variance $\sigma_{u}^{2}$, and truncated at zero; (i.e. $u_{h}∼N(|\mu_{h},\sigma_{u}^{2}|)$). In log form, Eq 2 can be written as:

$${log(Y}_{h})=\log\left\{f(X_{ih},\beta )\right\}+v_{h}-u_{h}$$ (2A)

The efficiency term assumes non-negative values since, by definition, it represents part of the distance between the actual output of a farm household and the maximum achievable output for a given set of inputs and technology. The gap between the actual output and frontier output is always non-negative as farms can never produce above the frontier output level. The technical efficiency index of farm household *h* (*TEI*_*h*_) can be computed as the proportion of actual production (*Y*_*h*_) relative to the maximum output achievable under perfect efficiency (i.e. frontier production level, $Y_{h}^{*}$). Thus, *TEI*_*h*_ can be given as:

$${TEI}_{h}=\frac{Y_{h}}{Y_{h}^{*}}=\frac{f(X_{ih},\beta )exp(v_{h}-u_{h})}{f(X_{ih},\beta )exp(v_{h})}{=e}^{-u_{h}}$$ (2B)

For farm households operating on the production frontier (i.e., perfectly efficient farm households), the value of *u*_*h*_ is zero (*u*_*h*_ = 0) and hence, their technical efficiency equals 1.

The classical SPFF in Eq 2 assumes that the dependent variable is normally distributed and the observations are independent. In our case, however, the latter assumption may be violated as the observations might be spatially correlated. For example, depending on the geographical location of the farm households (e.g., soil type, climate, and topography), the efficiency of the inputs that the farm households employ and the total production they achieve may be spatially correlated. Some areas may have a geographical advantage over others, and may be more favorable for agricultural production than others. Consequently, observations from similar locations may manifest a similar pattern implying spatial dependencies. To mitigate this problem, following the recent developments in SPFF, we used spatial stochastic production frontier analysis (SSPFA) [39].

In this case Eq 2A can be modified as:

$${log(Y}_{h})=\log\left\{f(X_{ih},\beta )\right\}+v_{h}-{\left(1-\rho \sum w_{h}\right)}^{-1}\hat{u_{h}}$$ (3)

Where $v_{h}∼N(0,\sigma_{v}^{2})$ and $u_{h}∼N(0,{\left(1-\rho \sum w_{h}\right)}^{-2}\sigma_{\hat{u}}^{2},$ *v*_*h*_ and *u*_*h*_ are distributed independently of each other and of the explanatory variables; $\hat{u_{h}}∼N(0,\sigma_{\hat{u}}^{2})$, *w*_*h*_ is a standardized matrix of spatial weights, and *ρ* the spatial lag parameter that measures the correlation between the efficiencies of neighboring observations.

#### Computation of the household assets included in our study

*Financial capital:* this category of capital includes the availability of cash or cash-equivalent assets [40]. The two main sources of financial capital are stocks (e.g., savings or livestock), and regular flows of money (e.g., regular cash income from remittance, pension and other sources) [23]. Generally, epidemics affect the financial capital of a household either through an increased expenditure (e.g., drug, medical treatment, and funeral ceremonies) or decreased access to the sources of such capital. As the EVD epidemic in Liberia represented a global health emergency, medical expenditures were mainly covered by the Liberian government and donations from the international community. Further, due to the highly infectious nature of EVD, public funeral ceremonies were rarely conducted. Thus, unlike other epidemics, such as malaria and HIV/AIDS, the adverse effect of the EVD epidemic on financial capital through increased expenditure may be less pronounced than through decreased access to the sources of financial capital. Therefore, in this study we focus on the effect of the EVD epidemic on the access to financial capital by Liberian households. The cash flows that the households obtained from different sources of financial capital were computed by asking the households to report the amount of cash income they received from pension, remittance and other sources in the 12 months prior to the survey.

The other type of financial capital used in our study is the ownership of livestock [23], which is one of the most important household assets in rural Africa [41–42]. Livestock can be used for transport, drawing ploughs, as well as milk, wool, meat and leather production. The different categories of livestock owned by sample households were converted into a tropical livestock unit (TLU) using the conversion factors suggested by FAO [43] (for details see S1 Appendix Table C).

*Natural capital:* This includes access to land and related assets owned by households [23, 44]. Due to the EVD epidemic, households may have been compelled to sell or rent out part of their farmland and other productive assets to cope with the economic stress caused by the epidemic. Further, households may have abandoned part of their farm land due to shortage of labor and/or cash. In this study, we used the possession of uncultivated farm land by a household as a proxy to investigate the effect of the EVD epidemic on natural capital.

*Social capital:* Although there is no single definition of social capital, it commonly refers to the social and cultural coherence of a society [45–46]. It plays a crucial role in improving economic efficiency by reducing transaction costs and increasing cooperation among the members of a society [47]. It also improves economic growth by fostering innovation [48].

Social capital is commonly explained in terms of trust [49–50]. In our study, trust is defined by two indices: trust in one’s community (sometimes known as interpersonal trust) [51], and institutional trust. These trust indices were obtained from Likert item responses to six trust related questions (for details see S5 Appendix). In our questionnaire, respondents were asked to rate their degree of agreement to different trust-related questions from 1 (strongly disagree) to 5 (strongly agree) and two indices for the social capital were obtained using a factor analysis [49, 51, 52]. The first one was the institutional trust index, which was composed of trust in village leaders and trust in the Liberian government. The second index was the interpersonal trust component, which included trust in the members of one’s community, tribe and religious associations, and the willingness of one’s community members to extend help when needed (see S5 Appendix). We also compared these results with the results obtained from an additive index approach (see S1 Appendix Table F) and found that the two approaches produced similar results. See Table 1 for the summary of the definition of the household assets included in our study.

### Methods of analysis

To analyze the data, we used descriptive statistics, a factor analysis and regression models. We used Stata 10.1 [53] and R 3.2.4 [54] for our analysis. In our analysis self-reported incidence of EVD in the community of the respondent was used as a proxy for EVD occurrence. Respondents were asked whether they knew anybody in their own or nearby communities who had contracted EVD. We used self-reported incidence of EVD in one’s community instead of EVD cases reported by WHO, as the publicly available WHO reports are at the spatial resolution of the county-level and not community-level. In our understanding, community-level incidence might be more relevant for household-level decisions, because the EVD incidence at the community spatial scale may have a larger impact on household livelihood than the EVD occurrence at the county spatial scale.

Our analysis is divided into two parts. First, we analyzed the effect of the EVD epidemic on livelihood outcomes, such as total annual household income and agricultural production, using descriptive statistics and a regression analysis. Second, we analyzed the impact of the epidemic on the households’ assets. Here, we used descriptive statistics to summarize the effect of the EVD epidemic on the resource bases of households, and a regression analysis to analyze changes in social capital. For the summary of the methods used see S1 Appendix Table A.

In our regression analysis, we employed spatial econometric models to account for potential spatial dependencies in the data using the “*spdep*” package in R [55]. Before we ran the models, we tested for the significance of the spatial correlation of the residuals obtained from the mixed-effects models (with random slopes and intercepts) using Moran’s test (see S1 Appendix Table D). The test results suggested that there was a significant spatial correlation in our data, justifying the use of spatial econometric models. We further checked for the suitability of spatial-lagged models and spatial-error models, but found no significant difference between these models. Hence, we report results from spatial-lagged models here and include the results from the spatial-error models in the appendix (see S1 Appendix Table E Models 3 and 6; and Table G model 3).

To estimate the effect of the EVD crisis on agricultural production, we used a spatial stochastic production frontier model with the “*ssfa*” package in R [39]. The application of the stochastic frontier model (instead of the classical linear regression model) was justified after testing for the significance of the existence of inefficiencies in our agricultural production data using the likelihood ratio test (LR test: chi-bar square (1) = 6.396, p = 0.006).

Finally, we conducted power analyses and found that the power of our tests ranged from 73–98%, indicating a relatively low probability of conducting Type II error.

## Results and discussion

### Characteristics of sample respondents

The majority of our respondents (90%) were male with an average age of 43 years. Almost 64% of the respondents were literate, which is comparable to 66.7% reported by LISGIS [56]. Respondents had attended school for an average of six years. Sample farm households owned, on average, 1.53 hectares of farm land, which is similar to the average farm size of 1.54 hectares reported by the FAO [57]. Forty-two percent of the sample respondents were from urban areas and 58% from rural areas (for more detail see S1 Appendix Table B). Thirty-one percent of the sample households reported the incidence of EVD in their community or nearby communities and almost 20% of these were households that relied heavily on farming for their livelihoods.

### The impact of EVD on livelihood outcomes

#### EVD epidemic and total household income

The annual average household income of the sample households was 153,322 Liberian Dollars (LRD) (equivalent to 1,825.6 USD) during the EVD crisis. We found no significant difference in the annual total income of households who reported the incidence of EVD in/near their community and those that reported no incidence of the epidemic in/near their community (n = 617, t = -0.771; p = 0.441). Nonetheless, more than half of the sample households reported that their income was lower during the EVD crisis than their income in the previous year. The decline could have been due to market interruptions and price fluctuations as a direct result of the EVD outbreak, and/or control measures imposed by Liberian government in an attempt to control the disease [15, 26, 58]. The widespread decline in household income across Liberia may suggest that the EVD crisis did not only affect those areas where people contracted and/or died of the disease, but also had an effect on the households’ income throughout the nation. These findings are supported by our regression analysis results (see Table 2), which revealed that there was no significant difference between the annual income of households in communities with and without the reported incidence of EVD. We also obtained similar results using matching techniques (t = 1.3; p = 0.2). (For the discussion of control variables please refer to S2 Appendix).

**Table 2:** Regression results for the impact of EVD on household income using spatial-lag models.

Variables^++^	Estimates	Sd. errors
**Test predictor variable**		
Incidence of EVD in the community^¥^: yes	0.137	0.113
**Control variables**		
Farm size (ha)	0.028	0.029
Residence: urban	-0.132	0.106
Rural (reference category)	n.a	n.a
Household size	0.004	0.013
Gender of household head: male	0.741***	0.173
Female (reference category)	n.a	
Age of household head (years)	-0.006	0.004
Education level of household head (years)	0.026***	0.01
Occupation of household head: formal employment	0.854***	0.140
Informal employment	0.5378***	0.152
Self-employment	0.44***	0.145
Skilled laborer	0.365	0.25
Farmer (reference category)	n.a	n.a
Constant	7.875***	0.628
Observations	501	501

Note: ^**++**^The dependent variable is the logarithm of total household income

^¥^ This is a dummy variable that assumes 1 if a household reports that they know of a person in their or neighboring community who contracted EVD, and 0 otherwise

*p<0.1

**p<0.05

***p<0.01.

#### EVD epidemic and agricultural production

The two major crops produced by sample households were rice (89%) and cassava (32%). Almost 82% of the farm households reported that they were still working on their farm during our survey. Rural households primarily earned their annual income from agricultural production (83%). The remaining 5% and 12% of their total annual income came from off-farm employment and other income sources such as remittance, gift and aid, respectively. Nearly 54% of the respondents reported that their agricultural production had decreased during the EVD epidemic, compared to their previous year’s production (i.e., before the outbreak). This reduction in production could be due to a decrease in the size of cultivable farm triggered by the closing of markets and lack of middlemen who purchase agricultural products from farm gates and transport them to the market centers [26]. However, we did not find a statistically significant difference in the proportion of sample households from communities with and without the incidence of EVD that reported a decrease in their agricultural production (Pearson χ^2^ (1) = 0.77, p = 0.378). A statistically similar proportion of sample respondents from communities where EVD occurred and did not occur reported a decrease in agricultural production.

The regression results using the SSPF model indicated that respondents who reported the incidence of EVD in/near their community produced significantly less crops during the crisis when compared to respondents who reported no incidence of EVD in their or neighboring community (see Table 3). Our results revealed that although most farm households from both communities, where EVD occurred and did not occur, reported that their production was lower compared to that of the previous year, the agricultural production of farm households from communities with the incidence of EVD was lower than the agricultural production of households in communities where EVD did not occur. (For the discussion of control variables please refer to S3 Appendix).

**Table 3:** Determinants of agricultural production using SSFM.

Variables^++^	Estimates	Sd. errors
**Test predictor variable**		
Incidence of EVD in the community ^¥^: yes	-0.534***	0.188
**Control variables**		
Ln (farm size)	0.595***	0.085
Ln (labor cost)	0.084***	0.023
Ln (number of male adults)	0.096	0.125
Ln (number of female adults)	-0.305**	0.149
Ln (education level of household head)	0.033	0.066
Ln (age of household head)	0.02	0.257
Household head gender: male	0.306	0.295
Constant	9.806***	1.111
N	311	
$\sigma_{u}^{2}\_\mathrm{dmu}$	0.858	0.841
$\sigma_{v}^{2}$	1.083***	0.315
*σ*^2^	1.228	
*λ*	0.533	
Moran I statistic	-0.061	
Mean efficiency	0.574	
Spatial parameter, *ρ*	0.008	
LR-test	7.812***	

Note: ^**++**^The dependent variable is the logarithm of the value of crop production at gross margin per hectare

^¥^ This is a dummy variable that assumes 1 if a household reports that they know of a person in their or neighboring community who contracted EVD, and 0 otherwise

*p<0.1

**p<0.05

***p<0.01.

The efficiency analysis showed that during the EVD crisis, surveyed households had an average efficiency level of 57.4%, though there was no significant difference between the average efficiencies achieved by farm households in communities with and without the reported incidence of EVD (t = -0.059; p = 0.953). Our findings suggest that although the incidence of EVD in a community had no significant effect on the technical efficiency of farm households, it had a significant effect on their total crop production during the EVD epidemic in Liberia.

### The impact of EVD on different categories of the households’ capital

#### EVD and financial capital

The annual financial capital obtained by sample households in terms of financial inflows during the EVD epidemic was 18,905 LRD (or 225.5 USD). The major share of the financial capital was obtained from gifts, remittances, aid and other sources. We found no significant difference in the annual financial income obtained by households from communities with and without the incidence of EVD (Mann-Whitney test: n = 149; z = 1.77; p = 0.076).

We also used the amount of livestock owned by households as a proxy for their financial capital (i.e., the stock part of the financial capital). Almost 62% of our sample households owned one or more categories of livestock. The sample households that reared livestock mainly owned chickens (82%), goats (36%) and sheep (16%). Only 6% of the sample households with livestock reported that they owned cattle. The average livestock ownership of the sample households who reported owning one or more livestock species was 1.26 TLU (median = 0.84). Households in communities where EVD occurred, owned significantly less livestock compared to sample households in communities with no incidence of EVD (Mann-Whitney test: n = 375; z = 2.48; p = 0.013). These results should be interpreted with caution, as the most affected parts of the country were generally less known for livestock production [59], which may have had a confounding effect on our results. Almost 47% of the households that owned livestock reported that the amount of their livestock decreased during the EVD epidemic compared to the year before the epidemic. This may be because respondents may have used livestock for their own consumption as the EVD crisis resulted in food shortages [35]. The decrease in livestock almost certainly affected the livelihoods of rural households in Liberia [59], as it does in other parts of rural Africa [42].

#### EVD and natural capital

To investigate the impact of the Ebola crisis on the natural capital of sample households, we compared the likelihood of leaving farm land uncultivated during the EVD epidemic by households from communities with and without the EVD incidence. In our sample, almost 21% of rural households reported that they had uncultivated farm land in the 12 months preceding the survey. Previous studies also reported that there was widespread reduction of cultivable farm size in Liberia during the Ebola crises [19, 20, 26, 58]. However, our study showed that the incidence of EVD in the community of the respondents did not have a significant effect on the likelihood of leaving farm land uncultivated (Pearson χ^2^ (1) = 1.709, p = 0.425). Most of the respondents who reported having uncultivated farm land (47%), stated that the main reason for not cultivating their farm was a lack of labor and money to buy farm inputs. Only 21% of the respondents attributed (at least partly) the existence of uncultivated farm land that they owned to the EVD epidemic. Nonetheless, the shortage of labor might be an indirect effect of the EVD epidemic, as collective work on agricultural fields was banned in response to the epidemic [15, 26].

#### EVD and social capital

The incidence of EVD had a significant effect on the trust of the respondents in Liberian institutions (n = 622, z = 2.157, p = 0.031). Respondents who reported the incidence of EVD in/near their community trusted Liberian institutions significantly less than those who reported no incidence of EVD in/near their community (Pearson χ^2^ (1) = 7.554, p = 0.006). Our regression results in Table 3 also suggest that the EVD epidemic negatively and significantly affected the respondents’ trust in Liberian institutions (Table 4 model 1). This may have broad and long-term implications for the stability of the political system [60] and economic growth of the country [48]. Almost 45% of the respondents reported that their trust in the Liberian government had declined during the EVD epidemic. These results support the findings of Tsai et al (2015) [27], who reported that during the EVD epidemic, trust in Liberian institutions, including the government, deteriorated. Furthermore, we found that almost 42% of our respondents reported that their trust in the village chief(s) declined during the EVD epidemic. The likelihood of reporting a decline in the trust in village chiefs was significantly higher for respondents from communities with the incidence of EVD, compared to respondents from communities with no incidence of the disease (Pearson χ^2^ (1) = 9.170, p = 0.002). On the other hand, we found no significant effect of the EVD incidence on the respondents’ trust in their community members (n = 622, z = 1.624, p = 0.104). These results were also corroborated by the regression analysis (Table 4 model 2). Almost 65% of the respondents reported that their trust in their community was not affected by the EVD epidemic. Only about 9% of the respondents suggested that their trust in their community had declined during the EVD epidemic. Men were found to have more trust in their community than women, which is consistent with the existing literature that demonstrates that men are generally more trusting than women in terms of interpersonal trust [61, 62].

**Table 4:** Regression results for the impact of EVD on social capital using spatial-lag models.

	Model (1) Trust in institutions		Model (2) Trust in community	
Variables	Estimates	Sd. errors	Estimates	Sd. errors
**Test predictor variable**				
Incidence of EVD in the community ^¥^: yes	-0.250**	0.107	0.104	0.112
**Control variables**				
Residence: urban	-0.573***	0.103	-0.180*	0.107
Religion: muslim	-0.134	0.154	0.026	0.161
Religion: other	0.07	0.279	-0.027	0.291
Religion: christian (reference)	n.a	n.a	n.a	n.a
Ln (total income)	0.019	0.019	0.020	0.019
Gender: male	0.154	0.130	0.436***	0.136
Education level (years)	-0.028***	0.0075	0.004	0.008
Age	0.005	0.0031	0.0001	0.003
Constant	-0.187	0.293	-0.395	0.305
Observations	553	553	553	553

Note: ^¥^ This is a dummy variable that assumes 1 if a household reports that they know of a person in their or neighboring community who contracted EVD, and 0 otherwise

*p<0.1

**p<0.05

***p<0.01

### Conclusion

Our study offers important insights on the effect of the EVD epidemic on the livelihoods of Liberian society and the mechanisms underlying them. We found that the incidence of EVD did not influence the total annual household income depending on whether or not the households were located in/near communities where EVD occurred. However, the majority of the sample households reported that their income was lower during the EVD crisis, as compared to their income before the outbreak. This suggests that the effects of the EVD epidemic were not limited to the communities where EVD occurred, but that the EVD crisis affected communities throughout the country. These results are in line with the findings of Bowles et al. (2016) [63] who reported that during the EVD epidemic, there was a remarkable decline in economic activities across Liberia, but that in most cases, there was little association between the decline in economic activities and the number of Ebola cases. Thus, post-epidemic rehabilitation measures should not only be limited to communities directly affected by EVD, but should also target those indirectly affected by the epidemic. We also found that the incidence of Ebola significantly reduced total crop production of farm households, which is in line with other studies [see also 15, 26, 58, 64]. As farm households in our sample had consumed about 85% of their own production, they heavily depended on their own agricultural production for survival, which is a typical characteristic of subsistence farmers. Thus, the reduction in their agricultural production likely had an adverse effect on their food security, which is in line with the existing literature [15, 20, 26, 35, 58, 64]. Most of these studies reported that the agricultural sector was one of the most severely affected sectors by EVD, and food security was significantly hampered by the epidemic in Liberia.

Our results also revealed that the incidence of EVD negatively affected the trust of the citizens in Liberian institutions. Respondents who reported the incidence of EVD in/near their community, were more likely to report a decrease in their trust in the government and village chief(s). Our results suggest that in the long term, the deterioration in the social capital resulting from the EVD epidemic, may have adverse effects on the stability of the country’s political system [60]. Degradation of social capital may increase the likelihood of social conflict and crime. Moreover, distrust in state institutions may render recovery efforts more challenging and make the country more susceptible to future outbreaks, as citizens may no longer comply with the recommendations of the state institutions [27].

Finally, although we controlled for most of the relevant factors in our analysis, there may still be some confounding effects, as our results were based on cross-sectional data collected at a single point in time. In addition, as it is customary to the surveys of our type, there could be limitations associated with retrospective memory, as respondents may not have accurately recalled information from the previous year, though we believe that one year is short enough for respondents to accurately report the events in their household. We believe that our results could help Liberia and other countries in the developing world with similar socioeconomic conditions, as well as the international community, to be better prepared for future crises and distribute livelihood rehabilitation efforts more effectively, thereby facilitating the affected nation’s speedy recovery after such crises.

## Supporting information

S1 Appendix Additional statistical analysis and discussions. (PDF)

S2 Appendix Discussion of control variables in Table 2. (PDF)

S3 Appendix Discussion of control variables in Table 3. (PDF)

S4 Appendix Credit access and EVD epidemic. (PDF)

S5 Appendix Social capital related questions in the questionnaire. (PDF)

S6 Appendix Questionnaire. (PDF)

S7 Appendix Memorandum of understanding and data sharing agreement between the researches’ institute and FDA, Liberia. (PDF)
