# Supplementary material for: The impact of the Ebola virus disease (EVD) epidemic on agricultural production and livelihoods in Liberia
Source: PLoS Negl Trop Dis. 2018 Aug 2;12(8):e0006580. doi: 10.1371/journal.pntd.0006580 (PMC6071957; doi:10.1371/journal.pntd.0006580)
Supplement: S1 Appendix — (PDF) [file pntd.0006580.s001.pdf]

Supporting information

S1 Appendix: Additional statistical analysis and discussions

Table A. The summary of methods used to analyze the data

	Analysis used	Models	R Package
**The effect of EVD on the livelihood outcome (part I)**			
Total household income	Both descriptive statistics and regression analysis	Spatial-lagged models and spatial-error models	spdep
Agricultural production	Both descriptive statistics and regression analysis	Spatial stochastic production frontier model	ssfa
**The effect of EVD on Resource base (part II)**			
Financial capital	Descriptive statistics		
Physical capital	Descriptive statistics		
Natural capital	Descriptive statistics		
Social capital	Both descriptive statistics and regression analysis	Spatial-lagged models and	spdep

		spatial-error models	

Table B: Descriptive statistics

Variable	Mean	Std. Dev.	Min	Max
Incidence of EVD in the community: Yes	0.311	0.463	0	1
Age of the respondent	42.29	12.416	18	85
Household size	8.424	3.993	1	18
Farm size (hectares)	1.532	1.946	0	25
Urban	0.42	0.491	0	1
Gender	0.902	0.297	0	1
Education level	6.184	5.255	0	18

Table C: Conversion factors for different categories of livestock

Camels	1.00
Cattle	0.70
Sheep	0.10
Goats	0.10
Horses	0.80
Mules	0.70
Asses	0.50
Pigs	0.20
Chicken	0.01

Source: Jahnke (1982)

Table D: Significance test for spatial correlation of the residuals obtained from Mixed-effect models

Dependent variable	Moran's I	SD	p-value
Community trust (Table 1)	0.1771	4.8965	0.000
Institutional trust (Table 1)	0.1297	3.5986	0.00016
Total Income (Table 2)	0.087	2.4506	0.00713

Table E: EVD and Social capital: Alternative models

	**Community Trust**			**Institutional Trust**		
	Model (1) Linear mixed-effects	Model (2) Linear model with Gaussian spatial correlation	Model (3) Spatial error Model	Model (4) Linear mixed-effects	Model (5) Linear model with Gaussian spatial correlation	Model (6) Spatial error Model
Incidence of Ebla in the community: Yes	0.0638	0.0401	0.0966	-0.1989*	-0.3646***	-0.2781***
	0.1142)	(0.1114)	(0.1121)	(0.1152)	(0.1084)	(0.1029)
Residence: Urban	0.2880	-0.1413	-0.1778*	-0.4709***	-0.6268***	-0.6158***
	(0.3606)	(0.1069)	(0.1061)	(0.1332)	(0.1038)	(0.0945)
Religion: Muslim	0.0019	0.0125	0.0220	-0.1081	-0.1412	-0.0888
	(0.1667)	(0.1601)	(0.1614)	(0.1625)	(0.1546)	(0.1482)
Religion: Other	0.0175	0.0446	-0.0368	0.1179	0.0302	0.1335

	(0.2893)	(0.2893)	(0.2960)	(0.2925)	(0.2792)	(0.2811)
Religion: Christian (reference)						
Ln (Total Income)	0.0064	0.0258	0.0215	0.0039	0.0030	0.0215
	(0.0196)	(0.0193)	(0.0194)	(0.0197)	(0.0186)	(0.0178)
Gender: Male	0.5087**	0.4386***	0.4434***	0.0580	0.2507*	0.2128
	(0.2427)	(0.1351)	(0.1381)	(0.1767)	(0.1309)	(0.1306)
Education level (years)	0.0127	0.0042	0.0046	-0.0245***	-0.0282***	-0.0308***
	(0.0079)	(0.0078)	(0.0081)	(0.0080)	(0.0076)	(0.0078)
Age	0.0031	0.0003	-0.0001	0.0032	0.0066**	0.0061**
	(0.0032)	(0.0032)	(0.0033)	(0.0032)	(0.0031)	(0.0031)
ac.term		-1.9025***			-1.6651***	
		(0.2071)			(0.1874)	
Constant	-1.0808**	-0.4444	-0.4217	0.1764	-0.1147	-0.2683
	(0.4821)	(0.3030)	(0.2996)	(0.4192)	(0.2926)	(0.2678)
N	553	553	553	553	553	553
R2		0.3272			0.3982	
Adjusted R2		0.2359			0.3165	

Log Likelihood	-712.3715		-704.0825	-708.3407		-671.7219
Akaike Inf. Crit.	1,570.7430		1,544.1650	1,562.6810		1,479.4440
Bayesian Inf. Crit.	1,885.7640			1,877.7030		
Residual Std. Error (df = 486)		0.8535			0.8241	
F Statistic (df = 66; 486)		3.5815***			4.8731***	
Wald Test (df = 1)			3.9353**			33.3193***
LR Test (df = 1)			3.0557*			23.8147***

Note: *p<0.1; **p<0.05; ***p<0.01

Table F: EVD and Institutional trust: Additive index

	Dependent variable: Institutional trust		
	Model (1) Complete sample	Model (2) Rural subsample	Model (3) Urban subsample
Incidence of EVD in the community: Yes	-0.0389**	-0.0073	-0.0705***
	(0.0187)	(0.0317)	(0.0233)
Residence: Urban	-0.1090***		
	(0.0180)		

Religion: Muslim	-0.0128	0.0320	-0.0332
	(0.0269)	(0.0348)	(0.0380)
Religion: Other	-0.0138	0.0472	-0.0184
	(0.0485)	(0.1174)	(0.0557)
Religion: Christian (reference)			
Ln (Total Income)	0.0016	0.0055	-0.0048
	(0.0032)	(0.0042)	(0.0045)
Gender: Male	0.0366	0.0673*	0.0230
	(0.0227)	(0.0344)	(0.0289)
Education level (years)	-0.0041***	-0.0026	-0.0032*
	(0.0013)	(0.0018)	(0.0017)
Age	0.0009*	0.0021***	0.0005
	(0.0005)	(0.0007)	(0.0007)
Constant	0.9701***	0.9627***	1.0154***
	(0.0731)	(0.1046)	(0.0891)
Observations	553	210	343
Log Likelihood	288.3293	168.0857	160.0758
sigma2	0.0205	0.0115	0.0227
Akaike Inf. Crit.	-440.6586	-230.1714	-222.1517
Wald Test (df = 1)	6.1287**	10.7684***	9.1232***
LR Test (df = 1)	6.0281**	10.1279***	8.0619***

Note: *p<0.1; **p<0.05; ***p<0.01

Table G: EVD and total household income: Alternative models

**Dependent variable: Logarithm of annual household Income**			
	Model 1: Linear mixed-effects	Model 2: Linear model with Gaussian spatial correlation	Model 3: Spatial error
Incidence of Ebola in the community: Yes	0.0658	0.1036	0.0994
	(0.1210)	(0.1136)	(0.1211)
Farm size	0.0601**	0.0402	0.0405
	(0.0297)	(0.0293)	(0.0294)
Residence: Urban	-0.0646	-0.1595	-0.1589
	(0.1224)	(0.1072)	(0.1169)
Household size	0.0045	0.0055	0.0052
	(0.0123)	(0.0124)	(0.0124)
Gender: Male	0.5912***	0.5755***	0.5626***
	(0.1839)	(0.1678)	(0.1674)
Age (years)	-0.0052	-0.0043	-0.0047

	(0.0040)	(0.0041)	(0.0040)
Education level (years)	0.0228**	0.0230**	0.0225**
	(0.0100)	(0.0100)	(0.0099)
Occupation: Farmer	-0.5437***	-0.5492***	-0.5426***
	(0.1731)	(0.1739)	(0.1725)
Occupation: Wage employment	0.3419*	0.3256*	0.3345*
	(0.1916)	(0.1927)	(0.1891)
Occupation: Informal employment	0.1202	0.1113	0.1166
	(0.2503)	(0.2458)	(0.2476)
Occupation: skilled labor	0.0687	0.1324	0.1835
	(0.2701)	(0.2723)	(0.2687)
Occupation: Small business	-0.5139**	-0.4722**	-0.4503**
	(0.2114)	(0.2137)	(0.2118)
Occupation: Others (reference)	NA	NA	NA
ac.term		0.4432***	
		(0.0866)	
Constant	10.9423***	10.9679***	10.9955***
	(0.3135)	(0.2971)	(0.2998)

Observations	528	528	528
R2		0.2104	
Adjusted R2		0.1904	
Log Likelihood	-813.1591		-793.7262
sigma2			1.1688
Akaike Inf. Crit.	1,666.3180		1,617.4520
Bayesian Inf. Crit.	1,751.7000		
Residual Std. Error		1.0910 (df = 514)	
F Statistic		10.5328*** (df = 13; 514)	
Wald Test			17.0610*** (df = 1)
LR Test			14.9260*** (df = 1)
Note:	*p<0.1; **p<0.05; ***p<0.01		

Table H: Determinants of agricultural production (spatial stochastic frontier model vs OLS)

Dependent variable: Ln (Value of crop production at gross margin per hectare)		
	SSFA Model	Ordinary Least squares (OLS) model
Incidence of EVD in the community: Yes	-0.534***	-0.5447***
	(0.188)	(0.1855)
Ln (Farm size)	0.595***	0.3962***
	(0.085)	(0.0857)
Ln (Labor cost)	0.084***	0.0701***
	(0.023)	(0.0236)

Ln (Number of male adult)	0.096	0.1196
	(0.125)	(0.1315)
Ln (Number of female adult)	-0.305**	-0.3495**
	(0.149)	(0.1528)
Ln (Education level of household head)	0.033	0.0450
	(0.066)	(0.0680)
Ln (Age of household head)	0.0199	0.1485
	(0.257)	(0.2748)
Household head gender: Male	0.306	0.3006
	(0.295)	(0.3009)
Constant	9.806***	8.5480***
	(1.111)	(1.0966)
N	311	311
σ_u^2_dmu	0.858	n.a
	(0.841)	
σ_v^2	1.0833***	n.a
	(0.315)	
σ^2	1.228	n.a
λ	0.5334	n.a
Moran I statistic	-0.0613	n.a
Mean efficiency	0.574	n.a
Spatial parameter, ρ	0.008	n.a

LR-Test	7.812; p=0.003	n.a
R2	n.a	0.1534
F Statistic	n.a	6.7948***(df= 8; 300)

Note: Standard errors in parenthesis; *p<0.1; **p<0.05; ***p<0.01.
