# Supplementary material for: The impact of the Ebola virus disease (EVD) epidemic on agricultural production and livelihoods in Liberia
Source: PLoS Negl Trop Dis. 2018 Aug 2;12(8):e0006580. doi: 10.1371/journal.pntd.0006580 (PMC6071957; doi:10.1371/journal.pntd.0006580)
Supplement: S2 Appendix — (PDF) [file pntd.0006580.s002.pdf]

S2 Appendix: Social capital related questions in the questionnaire

How much do you agree with the following statements?

Statement:	Strongly agree	Agree	Neutral	Disagree	Strongly disagree
Most of the people in my community are trustworthy					
Most of the people in my community are willing to help if needed					
Most of the people in my tribe are trustworthy					
Most of the people who belong to the same religion as I are trustworthy					
The government of Liberia is trustworthy					
The head of my community is trustworthy					
