# Supplementary material for: The impact of the Ebola virus disease (EVD) epidemic on agricultural production and livelihoods in Liberia
Source: PLoS Negl Trop Dis. 2018 Aug 2;12(8):e0006580. doi: 10.1371/journal.pntd.0006580 (PMC6071957; doi:10.1371/journal.pntd.0006580)
Supplement: S3 Appendix — (PDF) [file pntd.0006580.s003.pdf]

S3 Appendix: Discussion of control variables in Table 2

We also found that the gender of the household head had a significant effect on the annual income of the households, and households headed by men reported significantly higher annual income than those headed by women. In addition, households headed by more educated individuals reported significantly higher income than households headed by individuals with lower education level. Households headed by individuals with formal employment, informal employment, and self-employment had significantly higher income than households headed by individuals who identify themselves as farmers. On the other hand, there was no significant difference in the annual income of farm households and households whose heads reported skilled labor as their major source of livelihood. These results were robust across alternative models (see S1 Appendix Table E for results from alternative models).
