# Supplementary material for: The impact of the Ebola virus disease (EVD) epidemic on agricultural production and livelihoods in Liberia
Source: PLoS Negl Trop Dis. 2018 Aug 2;12(8):e0006580. doi: 10.1371/journal.pntd.0006580 (PMC6071957; doi:10.1371/journal.pntd.0006580)
Supplement: S4 Appendix — (PDF) [file pntd.0006580.s004.pdf]

S4 Appendix: Discussion of control variables in Table 3

Our results also showed that farm size had a significant influence on the amount of crop production of rural households in Liberia, as elsewhere in rural Africa [1]. Labor cost was positively and significantly associated with crop production, which may suggest that more investment in terms of hired labor leads to more agricultural production.[i] The other interesting finding was that the number of adult female members in a household was negatively associated with the agricultural production of the households. This may be due to the gender-based task allocation in rural Africa. In most societies in Africa, women are responsible for household tasks such as firewood collection, water fetching and child care [2, 3], which may reduce their time for agricultural production and other productive employment [3]. There could also be limited substitutability between men and women labor on some tasks [4].

Reference

1. Jayne, T. S., Yamano, T., Weber, M. T., Tschirley, D., Benfica, R., Chapoto, A., & Zulu, B. (2003). Smallholder income and land distribution in Africa: Implications for poverty reduction strategies. *Food Policy*, *28*(3), 253–275.
2. UNDP. (2011). *Human Development Report 2011: Sustainability and Equity: A Better Future for All*. *Sustainability and Equity: A better future for all*.
3. FAO; IFA& WFP. (2012). Rural Women and the Millennium Development Goals, 1–12.
4. Kabeer, N. (1994). Reversed realities: gender hierarchies in development thought. Verso.

[i] The labor cost includes only the amount paid for hired labor, and it does not include family labor.
