# Supplementary material for: The impact of the Ebola virus disease (EVD) epidemic on agricultural production and livelihoods in Liberia
Source: PLoS Negl Trop Dis. 2018 Aug 2;12(8):e0006580. doi: 10.1371/journal.pntd.0006580 (PMC6071957; doi:10.1371/journal.pntd.0006580)
Supplement: S5 Appendix — (PDF) [file pntd.0006580.s005.pdf]

S5 Appendix: Credit access and EVD epidemic

More than 40% of the sample respondents reported that they received credit from different sources such as microfinance, saving clubs, private lenders and others. The incidence of EVD in the respondents' community did not have a significant effect on the likelihood of taking loans (Pearson chi2(1) =1.8403; p = 0.175). In rural areas, however, the likelihood of taking loans was significantly influenced by the incidence of EVD in the respondents' community (Pearson chi2(1) = 4.8454; p=0.028). Households from rural communities where EVD occurred, were more likely to borrow during the EVD epidemic compared to households from communities without the incidence EVD. This may imply that rural respondents from communities with the incidence of EVD were filling the shortfall in their livelihoods by borrowing from different sources such as saving clubs, private lenders and others, which might be associated with the low rate of saving in rural areas. Urban households may live from their savings in such a crisis time and their likelihood of resorting to credit might be lower.
