# Supplementary material for: The impact of the Ebola virus disease (EVD) epidemic on agricultural production and livelihoods in Liberia
Source: PLoS Negl Trop Dis. 2018 Aug 2;12(8):e0006580. doi: 10.1371/journal.pntd.0006580 (PMC6071957; doi:10.1371/journal.pntd.0006580)
Supplement: S7 Appendix — (PDF) [file pntd.0006580.s007.pdf]

Max Planck Institute
for Evolutionary Anthropology

MEMORANDUM OF UNDERSTANDING

By and between

The project representatives, Prof. Dr. Hjalmar **KÜHL** and Jessica **JUNKER**, Max-Planck Institute for Evolutionary Anthropology (**MPIEVA**), Leipzig, Germany

And

The Forestry Development Authority of Liberia (**FDA**), Wheln Town, Liberia, represented by Hon. Kederick F. Johnson, Acting Managing Director of the FDA

December 2014

Preamble

This project aims to investigate underlying socio-economic drivers of hunting, bushmeat consumption, and meat preference across Liberia. We are particularly interested in local attitudes and behavioral changes in response to the Ebola crisis and whether the magnitude of these changes is influenced by socio-economic factors, such as literacy, ethnicity, religious belief, traditional taboos, education, and poverty, as well as law enforcement and availability of alternative protein sources, such as domestic animal meat. In addition, we would like to assess how and to what extent the Ebola outbreak impacted on people's livelihoods and economic well-being. With the information collected, we will be able to support the FDA in the implementation of effective evidence-based conservation management to protect Liberia's biodiversity and continue to provide human populations with the resources and ecological services they need.

- The **MPIEVA** and the **FDA** have a common objective, which is to contribute to the provision of scientific information that facilitates the effective protection of the environment, its biodiversity and ecosystem processes.
- The **MPIEVA** and **FDA** have collaborated since 2010 in biomonitoring of the entire country of Liberia.
- The **MPIEVA** and **FDA** have collaborated since 2010 in conducting socio-economic interview surveys in villages across the entire country of Liberia.
- The **MPIEVA** and **FDA** have collaborated since 2012 in establishing Temporary Research Sites in the East Nimba Nature Reserve and the proposed Grebo National Park to collect data on chimpanzee community structure and size, tool use, phenology and organic material.

I - Project

The present Memorandum of Understanding (MoU) covers the following project:
"The impact of Ebola on human socio-economic status and natural resource use"
The present MoU will last for phase 1 of the project, from 1. January 2015 to 31. May 2015, and phase 2, from 1. January 2016 to 31. May 2016, and is renewable by tacit agreement. These are preliminary dates, as the exact project duration will be strongly influenced by road condition, community collaboration, unforeseen logistical issues, etc. This MoU can be amended if need be by either party.

Contributions of the MPIEVA and FDA

The **MPIEVA** undertakes to:

- Provide all funding to execute this project including basic food, transport and lodging costs to **FDA** employees. (Refer to budget Annex 1)
- Provide additional funds on top of **FDA** monthly salaries to its employees for undertaking this research. (Refer to budget Annex 1)
- Provide scientific, technical and material support for the successful execution of this project. **MPIEVA** collaborates with FFI-Liberia to obtain material support for the project.
- Transfer capacities to **FDA** employees within the framework of this project.
- Engage in environmental education and Ebola awareness raising activities when visiting the different communities across the country for the purpose of conducting interviews.
- Communicate results of this project to **FDA** managers and senior technicians immediately after data collection has ceased and share both raw data and results of the statistical analyses, reports and publications with **FDA** (separate data sharing agreement already provided to FDA).
- Collaborate with national conservation agencies in Liberia and regularly update them on project progress and development.
- Provide safety rules outlining the risks of undertaking this work in an Ebola-affected country. Employees of the project must read, understand and agree to follow these rules by signing the document.

FDA undertakes to:

- Facilitate the **MPIEVA**'s work, including, but not exclusively for research permits and negotiation with local and national authorities,
- Provide a signed letter of introduction for the teams to present to the different county's (and district's) superintendents, government workers at road blocks and legal institutions, and chiefs and other authorities in local communities,
- Provide two employees to lead the survey teams as necessary for the smooth-running of the project.
- Ensure access to the parks and reserves and national forests of Liberia to the employees and collaborators of the **MPIEVA**
- Support the **MPIEVA** technically in their field activities where necessary
- Reiterate the risks taken by its employees in undertaking this project and ensures that the safety rules provided by the **MPIEVA** are read, understood, signed and adhered to.

II – Coordination, Evaluation, Data Sharing

Coordination

FDA agrees to provide the MPIEVA the following FDA employees and field team leaders (Menladi M. Lormie; Clement G. Tweh) for phase 1 (1. January 2015 to 31. May 2015) and phase 2 (1. January 2016 to 31. May 2016) this project to achieve the best coordination and execution of this nationwide project.

Evaluation

The contracted parties will meet once after phase 1 of the project has been completed and once the final stage (phase 2) of this project has been successfully completed; and any additional time required by either party

Data sharing

As stated and signed in a separate document (data sharing agreement.pdf) **MPIEVA** agrees to share all raw data collected during the 2010-2012, 2014/2015 and 2015/2016 Liberia nationwide interview surveys, and any reports, or publications resulting from this study with the **FDA**. **MPIEVA** further acknowledges **FDA's** participation and support in any grey literature report or published article resulting from this study.

Agreed upon

Leipzig, 09/12/2014
Prof. Dr. Hjalmar **KÜHL**

Project representative

Leipzig, 07-12-2014
Jessica **JUNKER**

Project representative

Monrovia,
Hon. Kederick F. Johnson

Acting Managing Director of the FDA
